# Application of artificial intelligence in postoperative orthopedic rehabilitation: a scoping review

**DOI:** 10.3389/fdgth.2025.1746552

**Published:** 2026-01-14

**Authors:** Jue Wang, Huihui Bi, Yawen Wang, Yixin Song, Hai Xu, Shenjie Zhong, Qiao He, Qiong Zhang

**Affiliations:** 1 School of Nursing, Zhejiang Chinese Medical University, Hangzhou, Zhejiang, China; 2 School of Public Health and Nursing, Hangzhou Normal University, Hangzhou, Zhejiang, China; 3 Department of Nursing, Zhejiang Provincial People’s Hospital, Hangzhou, Zhejiang, China

**Keywords:** artificial intelligence, orthopedic, postoperative, rehabilitation, scoping review

## Abstract

**Objectives:**

Artificial intelligence (AI) has shown increasing promise is orthopedic medicine. However, its role in postoperative rehabilitation remains insufficiently synthesized, particularly when rehabilitation is viewed as a continuous and dynamic care process. This scoping review aims to systematically map current AI applications in postoperative orthopedic rehabilitation, indentify prevailing application patterns and evidence gaps, and clarify their clinical and nursing implications.

**Methods:**

This scoping review was conducted following the methodological framework by Arksey and O’Malley. A comprehensive literature search was conducted in PubMed, CINAHL Complete, The Cochrane Library, Web of Science, Embase, Scopus, IEEE Xplore, SinoMed, China National Knowledge Infrastructure (CNKI), and the WanFang Database for studies published between March 2020 and March 2025. Data extraction and descriptive synthesis were performed on all included studies.

**Results:**

A total of 38 articles were included in this review, encompassing 3 core AI technologies, namely machine learning (ML), natural language processing (NLP), and expert systems (ES). These technologies were mainly applied in patients undergoing joint replacement, fracture repair, and spinal surgery, with the main application scenarios focusing on risk prediction, dynamic feedback, and rehabilitation monitoring. Notably, most studies focused on short-term predictive outcomes, while limited evidence addressed AI-supported intervention adjustment, nursing decision support, or long-term functional recovery.

**Conclusion:**

This review highlights that, despite rapid technological progress, AI in postoperative orthopedic rehabilitation remains largely predictive rather than interventional. The novelty of this review lies in its stage-oriented synthesis of AI applications across the rehabilitation continuum, revealing a critical gap between data-driven prediction and clinically actionable rehabilitation support. Future research should prioritize high-quality, longitudinal studies and shift toward AI-enabled preventive and adaptive rehabilitation strategies to facilitate meaningful clinical translation.

## 1 Introduction

Artificial intelligence (AI) refers to an integrated technological framework that simulates human intelligence through machines such as computers and robots, enabling functions including learning, reasoning, and problem-solving (1). Its major branches include machine learning (ML), computer vision (CV), fuzzy logic (FL), expert systems (ES), and natural language processing (NLP), among others. With the rapid development of algorithms, data resources, and computing power, AI has evolved from theoretical exploration to practical implementation, achieving remarkable success across diverse domains like medical diagnosis, smart manufacturing, and financial services (2–4). In recent years, the application of AI in healthcare has expanded continuously, especially within orthopedics, where it offers novel perspectives and innovative approaches for disease diagnosis, treatment planning, and rehabilitation (5). However, despite these advances, orthopedic clinical practice, especially rehabilitation continues to face significant challenges. Traditional rehabilitation approaches largely depend on subjective clinical judgment and experience, limiting their ability to meet patients’ individualized and precision rehabilitation needs (6). Owing to its powerful data-processing and pattern-recognition capabilities, AI can rapidly analyze medical imaging such as x-ray and CT scans (7, 8), highlighting its clinical feasibility. More importantly, AI enables efficient and accurate acquisition of patient data, thereby reducing clinicians’ workload and alleviating workforce shortages (9).

For patients undergoing orthopedic surgery, postoperative rehabilitation represents a crucial phase in restoring motor function, preventing complications, and reestablishing biomechanical homeostasis. The integration of AI with big data analytics provides new opportunities to optimize postoperative rehabilitation care by enabling predictive analytics (10), the developing of personalized training programs (11), and objective recognition and evaluation of patient movements (12). In nursing practice, AI-assisted rehabilitation systems can improve the objectivity and consistency of functional assessments, reduce the workload of healthcare professionals, and enhance patient compliance and safety during rehabilitation. Evidence-based research also indicates effective postoperative rehabilitation not only improves functional recovery and overall clinical outcomes but also contributes positively to patient's psychological well-being (13).

Although existing reviews have provided valuable insights into the application of artificial intelligence in orthopedics, current evidence remains fragmented with respect to the postoperative rehabilitation phase. Most prior studies (14–16) have primarily focused on preoperative diagnosis, surgical assistance, or outcome prediction, while the systematic role of AI in postoperative rehabilitation, particularly in rehabilitation process monitoring, personalized intervention design, functional assessment, and long-term recovery management has not been comprehensively synthesized.

From a clinical perspective, postoperative orthopedic rehabilitation is highly individualized, dynamic, and resource-intensive, yet conventional rehabilitation strategies still rely heavily on subjective clinical judgment and intermittent assessments, limiting their ability to provide continuous, precise, and adaptive care. From a research perspective, although diverse AI technologies such as machine learning based prediction models, computer vision–assisted motion analysis, and wearable sensor driven monitoring systems have been increasingly explored, there is a lack of integrative analysis linking specific AI techniques to distinct rehabilitation stages, clinical outcomes, and nursing practices. Moreover, the absence of a unified conceptual framework hampers the translation of AI-driven rehabilitation tools into routine clinical practice, making it difficult to evaluate their real-world effectiveness, safety, and feasibility. Therefore, a systematic mapping of current evidence is urgently needed to identify research trends, methodological limitations, and underexplored areas, as well as to clarify how AI can be optimally embedded into postoperative orthopedic rehabilitation pathways.

However, current evidence in this field is highly heterogeneous in terms of AI techniques, rehabilitation stages, outcome measures, and study designs, rendering conventional systematic reviews or meta-analyses focused on efficacy premature and methodologically inappropriate. A scoping review is a necessary and appropriate first step to map the breadth and structure of existing research, clarify key concepts, identify evidence gaps, and inform future study design. Scoping reviews are particularly suited to emerging and complex fields where evidence is fragmented and the primary aim is to establish an overarching knowledge framework rather than draw definitive conclusions on clinical effectiveness (17).

Accordingly, this study employs a scoping review methodology to systematically map the existing evidence on AI applications in postoperative orthopedic rehabilitation. The objectives are to summarize current technological approaches, evaluate their clinical and nursing-related implications, and establish a structured knowledge framework to support the development of precision, patient-centered rehabilitation strategies and guide future research directions.

## 2 Methods

### 2.1 Design and research question

This scoping review was conducted in accordance with the 5 steps framework proposed by Arksey and O’Malley (18), which comprises (1) identifying the research question, (2) identifying relevant studies, (3) selecting the studies, (4) charting the data, and (5) collating, summarizing, and reporting the results. The study protocol was registered on the Open Science Framework website (https://doi.org/10.17605/OSF.IO/QHT28). This scoping review, being secondary research with no new data collection or subject involvement, does not require ethical committee approval. In addition, the Preferred Reporting Items for systematic reviews and meta-analyses extension for scoping reviews (PRISMA-ScR) checklist was utilized for reporting (19). The filled PRISMA-ScR checklist is available in Supplementary File S1. The specific research questions that guided this review are as follows: (1) What types of AI technologies have been applied in postoperative orthopedic rehabilitation. (2) For which types of orthopedic surgeries have AI been primarily utilized in postoperative rehabilitation. (3) What are the functional applications and effects of AI in postoperative orthopedic rehabilitation, and in which aspects have its advantages and limitations been demonstrated.

### 2.2 Eligibility criteria

The inclusion criteria of this scoping review are as follows (1) Studies involving participants who had undergone orthopedic surgery. (2) Research focusing on the application of AI in postoperative rehabilitation management for orthopedic patients. (3) Original studies on the development and/or validation of AI tools, including model construction or usability testing. (4) Study designs comprising randomized controlled trials (RCTs), quasi-experimental studies, cohort studies, and case-control studies. Studies were excluded if the full text was unavailable, data were incomplete, or the publication was duplicated. Non-English and non-Chinese articles, as well as secondary literature such as reviews, meta-analyses, conference abstracts, editorials, animal experiments, and letters, were also excluded. In addition, studies that did not involve rigorous AI methodologies as those lacking substantive components of ML, or NLP were excluded.

### 2.3 Information sources and search strategy

The following databases were searched: PubMed, CINAHL Complete, The Cochrane Library, Web of Science, Embase, Scopus, IEEE Xplore, SinoMed, China National Knowledge Infrastructure (CNKI), and WanFang Database. The search period was from March 2020 to March 2025. An example of the search strategy used for Web of Science was as follows [TS = (“artificial intelligence” OR “data mining” OR “fuzzy logic” OR “neural networks, computer” OR “machine learning” OR “natural language processing” OR “computer reasoning” OR “machine intelligence” OR “computational intelligence” OR “computer vision systems” OR “computer vision system” OR “knowledge representation” OR “knowledge acquisition” OR “fuzzy algorithms” OR “neural network” OR “computer neural network” OR “neural network model” OR “computational neural network” OR “Bayesian networks” OR “text mining” OR “deep learning” OR “random forest” OR “support vector” OR “algorithms” OR “expert systems”)] AND [TS = (“orthopedics” OR “fracture” OR “arthroplasty, replacement” OR “arthroplasty” OR “joint” OR “hip” OR “knee” OR “shoulder” OR “ankle” OR “wrist” OR “elbow” OR “finger” OR “spine” OR “spinal” OR “vertebra” OR “skeleton” OR “dislocation” OR “subluxation” OR “trauma” OR “arthritis” OR “osteomyelitis” OR “osteoporosis” OR “ACL” OR “PCL” OR “bone tumor” OR “bone neoplasms”)] AND [TS = (“postoperative period” OR “postoperative care” OR “rehabilitation” OR “recovery of function” OR “postoperative periods” OR “postoperative procedures” OR “postoperative procedure” OR “function recoveries” OR “function recovery” OR “postoperative rehabilitation” OR “post-surgical recovery” OR “surgical recovery”)]. The details of retrieval search strategies were described in the Supplementary File S2.

### 2.4 Study selection

Retrieved studies were imported into EndNote 21 for deduplication. Guided by the established inclusion and exclusion criteria, two reviewers independently performed the initial screening of titles and abstracts. Following this, full-text studies were reviewed for secondary screening. Ultimately, the final set of studies for inclusion was finalized. Any discrepancies were resolved through discussion with a third reviewer.

### 2.5 Date extraction

Two reviewers independently extracted data using Microsoft Excel spreadsheets. The extracted information included: author, publication year, country, study design, types of surgery, sample, AI technology, application scenarios and functions, and key findings/results. If any disagreements exist between the two reviewers, they will be discussed with a third reviewer and finalized.

## 3 Results

**Figure 1:**
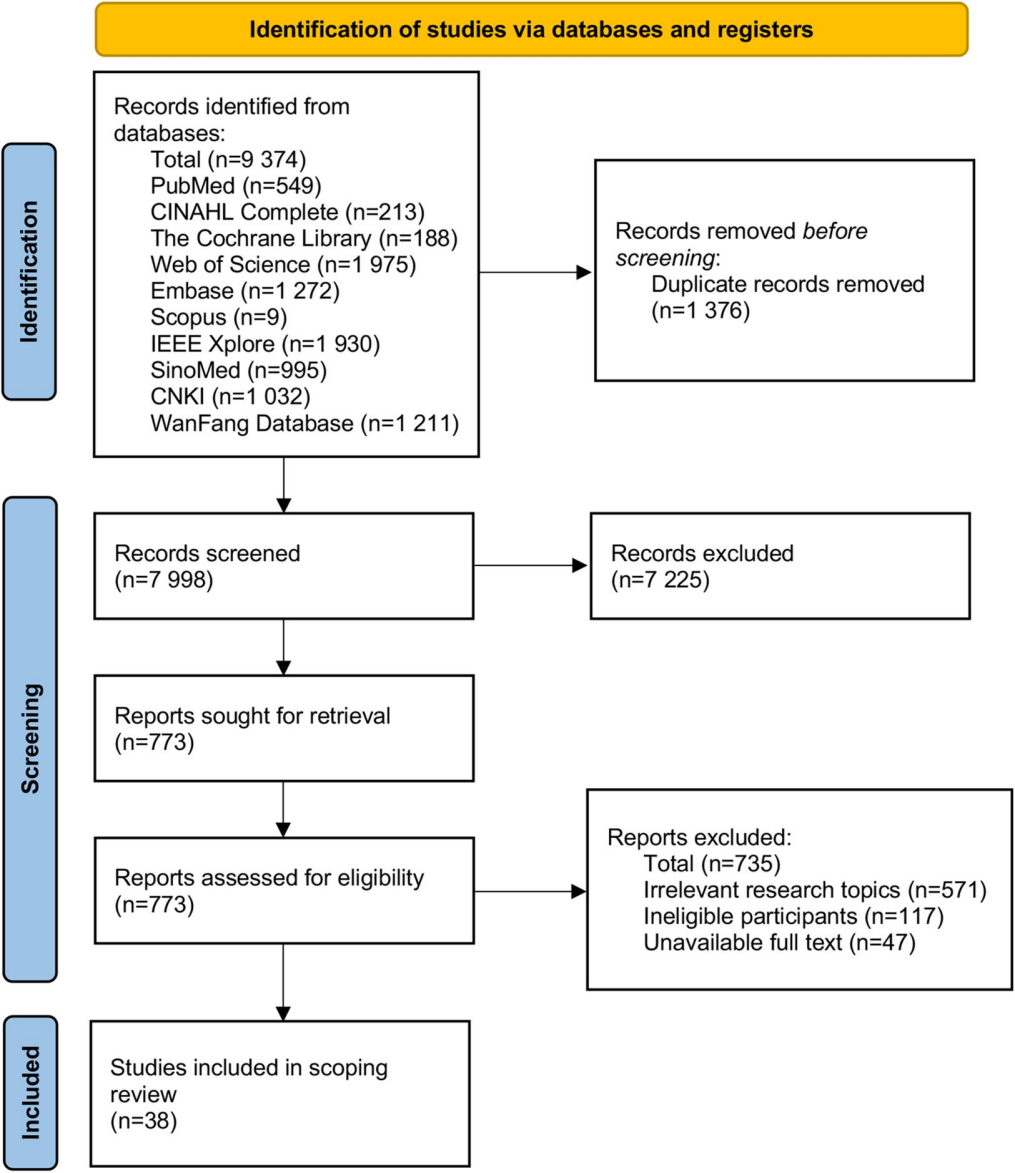
Flow chart of literature screening.

A total of 9,374 studies were retrieved. Following deduplication, 7,998 studies were screened by title, abstract, and full text, ultimately resulting in the inclusion of 38 articles. A PRISMA flow chart showing the study selection at each stage is detailed in Figure 1.

### 3.1 Characteristics of the included studies

A total of 38 articles were included, comprising 12 in Chinese and 26 in English publications. The distribution by country of publication was as follows, China (10–12, 20–36) (*n* = 20), USA (37–40) (*n* = 4), Korea (41–44) (*n* = 4), Canada (43, 45, 46) (*n* = 3), Iran (47) (*n* = 1), Japan (48, 49) (*n* = 2), France (50) (*n* = 1), Sweden (51) (*n* = 1), Spain (52) (*n* = 1), Australia (53) (*n* = 1). The study designs included retrospective cohort studies (11, 12, 25–28, 31, 33, 35, 41, 43, 48, 49, 51) (*n* = 19), prospective case-control studies (10, 20–22, 24, 32, 42) (*n* = 7), prospective cohort studies (44, 45, 53, 54) (*n* = 4), RCT (23, 29, 47, 52) (*n* = 4), quasi-experimental studies (30, 34) (*n* = 2), and validation studies (46, 50) (*n* = 2). Detailed information on the characteristics of the included studies was presented in Table 1.

**Table 1:** Characteristics of the included studies.

Author year country	Study design	Types of surgery	Sample	AI technology	Application scenarios and functions	Key findings/results
Chen et al., 2020 (20) China	Retrospective case-control study	Patellar fracture surgery	137	Support Vector Machine (SVM), logistic regression (LogR), LASSO regression	Prediction of sarcopenia risk	With an area under the curve (AUC) around 0.88, the prediction model exhibits favorable predictive performance and calibration, allowing for early detection of high-risk patients.
Wang et al., 2020 (21) China	Retrospective case-control study	Transforaminal lumbar interbody fusion (TLIF)	88	SVM	Prediction of cage subsidence risk	The Gaussian kernel SVM model was constructed. Sensitivity was 0.89, specificity was 0.97, and the AUC was 0.93.
Zhang et al., 2020 (37) USA	Retrospective cohort study	Thoracic/lumbar spine surgery	19,317	SVM, LogR, LASSO regression, ensemble learning (EL), artificial neural networks (ANNs)	Prediction of risk for long-term opioid use dependency	The key risk factors included preoperative high-dose opioid use, postoperative opioid dose escalation, and extended duration of therapy. Regression-based models performed best, accurately identifying around 75% of chronic users.
Chen et al., 2021 (22) China	Retrospective case-control study	Total knee arthroplasty (TKA)	634	SVM, LogR, EL	Prediction of blood transfusion requirement	The eXtreme Gradient Boosting (XGBoost) model outperformed others, with an AUC of 0.89 significantly surpassing alternative models, contributing to a reduction in unnecessary transfusions and complication risks.
Polus et al., 2021 (45) Canada	Prospective cohort study	Total hip arthroplasty (THA)	72	SVM, linear discriminant analysis (LDA), linear regression (LR), principle component analysis (PCA)	Prediction of fall risk	By combining preoperative and early postoperative data, the model attained an accuracy of 0.90, with sensitivity at 0.93, specificity at 0.58, and an AUC of 0.88, supporting the early formulation of rehabilitation plans.
Pierce et al., 2021 (38) USA	Retrospective cohort study	Corrective surgery for spinal deformity	9,143	ML	Prediction of postoperative complications and mortality	The predicted rates from the ACS-National Surgical Quality Improvement Program (ACS-NSQIP) tool were comparable to observed rates: 11.54% vs. 11.4% for overall complications, 10.48% vs. 10.1% for serious complications, and 0.28% vs. 0.9% for mortality. The near-zero Brier score indicates the tool has clinical applicability.
Liu et al., 2022 (10) China	Retrospective case-control study	Lumbar spine surgery	288	LogR, EL, ANNs, decision tree (DT)	Prediction of postoperative surgical site infection (SSI)	The XGBoost model emerged as the top-performing model, achieving a sensitivity of 0.83 and an AUC of 0.92. It correctly identified 86% of patients with SSI.
Wang et al., 2022 (23) China	RCT	THA	40/40	ANNs	Rehabilitation support encompassing personalized care and postoperative complication prediction	The intervention group showed significantly better outcomes than the control group: lower visual analogue scale (VAS) pain scores (1.17 vs. 3.05), higher Harris Hip Scores (79.47 vs. 67.26), improved Barthel Index scores (76.34 vs. 62.65), and reduced complication rates.
Liu et al., 2022 (24) China	Retrospective case-control study	TKA	100	EL	Prediction of deep vein thrombosis (DVT)	With an AUC of 0.83, this model facilitates the early identification and intervention for high-risk patients by clinicians.
Liao et al., 2023 (11) China	Retrospective cohort study	Anterior cruciate ligament reconstruction (ACLR)	15/15	ES	Rehabilitation support	The AI-Assisted telerehabilitation group showed significantly better international knee documentation committee (IKDC) and knee injury and osteoarthritis outcome score (KOOS) scores compared to the control group. This approach was associated with superior short-term efficacy and led to improved patient adherence.
Wang et al., 2023 (12) China	Retrospective cohort study	Arthroscopic surgery for rotator cuff tears	22/25	Virtual reality (VR), somatosensory interaction	Rehabilitation support	The telerehabilitation system (TR system) demonstrated significantly better shoulder function scores, improved range of motion and rehabilitation satisfaction, and effectively alleviated patient anxiety, depression, and pain compared to conventional rehabilitation.
Chen et al., 2023 (25) China	Retrospective cohort study	THA	476	SVM, LogR, EL, DT	Prediction of postoperative delirium	The model demonstrated a sensitivity of 0.85, a specificity of 0.90, and an AUC of 0.94, enabling the identification of high-risk individuals, thereby reducing the incidence rate.
Guo et al., 2023 (26) China	Retrospective cohort study	Hip fracture surgery	805	LogR, LASSO gression, EL, ANNs, DT, K-Nearest Neighbors (KNN)	Prediction of postoperative pneumonia	The model developed using the XGBoost algorithm exhibited remarkably high predictive performance, achieving an AUC exceeding 0.99.
Tsai et al., 2023 (27) China	Retrospective cohort study	TKA	3,495	LogR	Prediction of risk for long-term opioid use dependency	The prediction model, achieving an AUC of 0.75, enables the effective identification of patients at high risk for opioid dependence after orthopedic surgery.
Yan et al., 2023 (28) China	Retrospective cohort study	Internal fixation for femoral neck fracture	249	SVM, LogR, LASSO regression	Prediction of postoperative osteonecrosis of the femoral head (ONFH)	ML algorithms identified four postoperative risk variables. A nomogram prediction model was subsequently developed, achieving an AUC of 0.81.
Hua et al., 2023 (29) China	RCT	Internal fixation for double fractures of the tibia and fibula	40/40	Rule engine (RE)	Pain management	The implementation of the AI-PCA effectively enhanced postoperative analgesia and improved patient sleep quality, with both outcomes significantly lower in the intervention group than in the control group (*P* < 0.05). Additionally, patient satisfaction was higher (92.5% vs. 70.0%).
Yue et al., 2023 (30) China	Quasi-experimental studies	THA	150/150	NLP	Rehabilitation support encompassing health education, rehabilitation guidance, psychological support	AI technology can enhance patients’ health beliefs and self-management abilities, leading to a 6.66% reduction in secondary fracture incidence.
Wang et al., 2023 (31) China	Retrospective cohort study	Internal fixation surgery for hip fractures	80/80	RE	Pain management	The intervention group exhibited significantly lower postoperative pain scores, decreased adverse reaction rates, and higher patient satisfaction (95.0% vs. 82.5%) compared to the control group.
Rao et al., 2023 (32) China	Retrospective case-control study	Hip fracture surgery	130	LogR	Prediction of postoperative constipation	With an AUC of 0.94, the prediction model demonstrated excellent discriminatory ability.
Karabacak et al., 2023 (39) USA	Retrospective cohort study	Excision of spinal neoplasms	3,073	EL, KNN	Prediction of short-term postoperative adverse outcomes	A multi-outcome risk prediction model was developed, achieving an accuracy exceeding 0.75 and a mean AUC surpassing 0.70. This model was operationalized into a clinically applicable online tool to identify high-risk individuals.
Karabacak et al., 2023 (40) USA	Retrospective cohort study	Cervical disc arthroplasty (CDA)	6,604	EL, KNN	Prediction of short-term postoperative adverse outcomes	The prediction models achieved an overall accuracy of 0.88. Among them, the random forest (RF) model demonstrated the best performance, with an AUC of 0.83.
Shin et al., 2023 (41) Korea	Retrospective cohort study	Posterior cervical decompression surgery	59	Bayesian networks (BNs)	Prediction of neurological functional recovery	The model achieved an overall accuracy of 72.6%. For predicting postoperative Japanese Orthopaedic Association scores categorized as low, moderate, and high states, AUC values were 0.96, 0.85, and 0.79 respectively, enabling provision of personalized prognostic probabilities for patients.
Kazemnejad et al., 2023 (47) Iran	RCT	ACLR	10/10	ES	Rehabilitation support	The intervention group exhibited significantly lower mean postoperative VAS pain scores (1.20 vs. 3.60 in controls), enhanced functional and balance outcomes, and reported greater engagement and satisfaction regarding the exergaming intervention compared to the control group.
Fujii et al., 2023 (48) Japan	Retrospective cohort study	THA	343	LogR, EL, ANNs, DT	Prediction of changes in pelvic tilt	RF emerged as the top-performing model for predicting 5-year postoperative pelvic flexion angle changes, with an AUC of 0.852. To facilitate clinical application, a DT model was constructed to stratify patient risk, enabling accurate identification of those developing pelvic anteversion greater than 20° after surgery.
Boninn et al., 2023 (50) France	Validation study	TKA	22,759	ANNs	Automated analysis of postoperative imaging	The AI tool X-TKA demonstrated exceptional performance in image quality control and feature recognition, achieving a mean AUC of 0.98 and accuracy exceeding 95%. For anomaly detection, AI assistance provided surgeons with a 5% absolute improvement in accuracy and a 12% increase in sensitivity.
Dwyer et al., 2023 (54) Canada	Prospective cohort study	Arthroscopic surgery of the hip	26	NLP	Rehabilitation support encompassing pain management, wound care guidance, rehabilitation exercise reminders	The AI chatbot received “good” or higher ratings from 80% of patients. It effectively improved patient satisfaction and decreased healthcare resource use; however, enhancements are needed in semantic understanding and emergency response handling.
Wang et al., 2024 (33) China	Retrospective cohort study	Traumatic cervical spinal cord injury (SCI) surgery	315	EL	Prediction of motor functional recovery	With an accuracy of 0.81, the predictive model exhibited a low false negative rate, indicating strong performance in minimizing missed diagnoses.
Dai et al., 2024 (34) China	Quasi-experimental studies	SCI Surgery	45/43	ANNs	Rehabilitation support encompassing personalized rehabilitation care, risk prediction	The intervention group demonstrated significantly better outcomes in self-efficacy, activities of daily living, and quality of life compared to the control group.
Gan et al., 2024 (35) China	Retrospective cohort study	Total joint arthroplasty	1,800	SVM, LogR, EL, DT	Prediction of healthcare-associated infection risk	In developing risk prediction models with machine learning algorithms, XGBoost and RF emerged as superior performers in comprehensive evaluation across multiple metrics.
Hwang et al., 2024 (42) Korea	Retrospective case-control study	ACLR	102	SVM, LogR, EL, ANNs, DT	Prediction of motor recovery at 12 months postoperatively	RF demonstrated top performance in predicting both single-leg hop test outcomes and Tegner Activity Scores, while XGBoost models showed superior performance specifically for single-leg vertical jump test predictions.
Lee et al., 2024 (43) Korea	Retrospective cohort study	TKA	56/55/17	LogR, EL	Prediction of postoperative changes in gait speed	Preoperative gait velocity, age, and mechanical axis alignment were identified as key predictors. The model achieved high predictive accuracy, enabling healthcare providers to assess postoperative functional recovery with enhanced precision.
Park et al., 2024 (44) Korea	Prospective cohort study	Hip fracture surgery	132	EL, ANNs	Prediction of recovery trajectory	Based on acute-stage datasets, the predictive framework demonstrated robust performance with 72.2% classification accuracy and an AUC of 0.83.
Phellan et al., 2024 (46) Canada	Validation study	Scoliosis correction surgery	64	EL, ANNs, LR, PCA, DT, KNN	Prediction of postoperative spinal alignment	Integrating principal component analysis with LR yielded the optimal real-time prediction model. This solution achieved marginal prediction error at 3 ms inference time, excelling in accuracy and operational efficiency.
Morita et al., 2024 (49) Japan	Retrospective cohort study	THA	538	LogR, EL	Prediction of postoperative bone loss	With an AUC of 0.73 and Brier score of 0.20, the predictive framework enabled computational simulation of the preventive effect of bisphosphonates on bone-loss, thereby informing optimal medication strategies in clinical practice.
Blasco et al., 2024 (52) Spain	RCT	Reverse total shoulder arthroplasty (RTSA)	17/14	DT	Enhancement of early postoperative home rehabilitation adherence	The experimental group exhibited superior outcomes relative to controls: higher adherence rates (77% vs. 65%), more substantial Quick version of the Disabilities of Arm, Shoulder and Hand Questionnaire (QuickDASH) score improvements.
Xu et al., 2025 (36) China	Retrospective cohort study	Internal fixation of humeral fractures	275	SVM, LogR, EL	Prediction of secondary screw cut-out	The model achieved a sensitivity of 0.80, specificity of 0.77, and an AUC of 0.87, while the calibration curve demonstrated strong concordance between predicted and observed risks, and decision curve analysis indicated favorable clinical utility.
Buwaider et al., 2025 (51) Sweden	Retrospective cohort study	Anterior cervical discectomy and fusion (ACDF)	2,708	LogR, EL, KNN	Prediction of risk for persistent postoperative dysphonia	RF emerged as a robust algorithm for identifying persistent dysphonia after surgery, demonstrating a sensitivity of 0.89, specificity of 0.20, and an AUC of 0.79.
Ribbons et al., 2025 (53) Australia	Prospective cohort study	TKA	863	LR, DT, Bayesian inference (BI)	Prediction of functional improvement status	A prediction model incorporating biopsychosocial factors was developed, demonstrating robust predictive performance for quality of life enhancement and knee symptom improvement, enabling identification of high-risk cohorts and guidance of evidence-based rehabilitation planning.

### 3.2 Types of AI technologies employed

The AI technologies employed in the included studies primarily encompass a variety of machine learning and related intelligent methods. Among them, traditional ML algorithms were the most widely employed, such as support vector machine (SVM), logistic regression (LogR), and LASSO regression. These methods were often utilized to develop risk prediction and classification models (20, 28, 37). Furthermore, ensemble learning (EL) methods, including random forest (RF) and gradient boosting algorithms, were adopted in multiple studies to handle multivariate data and perform outcome prediction (37, 51). Compared with standard rehabilitation, artificial neural networks (ANNs), rule engine (RE) and ES have demonstrated better clinical applicability and patient satisfaction in pain management and improvement of rehabilitation compliance (11, 23, 29, 37). Some studies also utilized algorithms like decision tree (DT), k-nearest neighbors (KNN), and Bayesian networks (BNs) to achieve different forms of prediction or decision support (40–42, 51–53). In addition to numerical modeling approaches, NLP, and intelligent systems incorporating virtual reality (VR) or somatosensory interaction were also applied in relevant studies (12, 30, 54).

### 3.3 Types of surgery in AI applications

**Figure 2:**
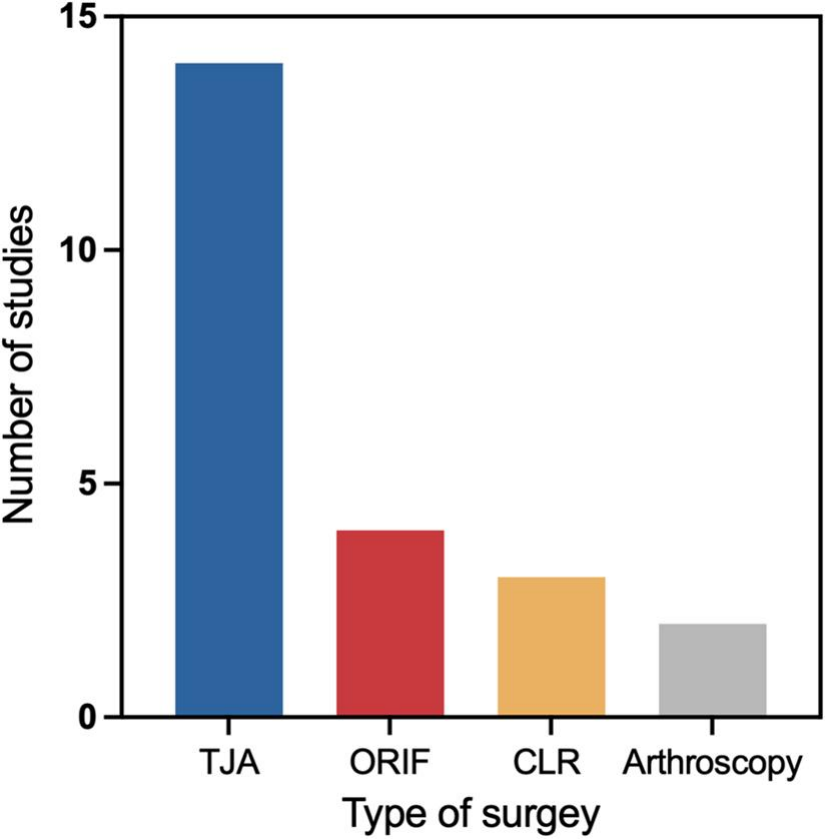
Number of included studies by surgical type.

The included studies encompass seven primary categories of surgical types. Among these, total joint arthroplasty (TJA) accounts for the largest proportion (*n* = 14) (22–25, 27, 30, 35, 43, 45, 48–50, 52, 53), including hip and knee arthroplasties. This is followed by open reduction and internal fixation (ORIF), which represents the second most commonly investigated surgical approach (*n* = 4) (23, 24, 26, 31). In contrast, cruciate ligament reconstruction (CLR) (*n* = 3) (11, 42, 47) and arthroscopic surgery (*n* = 2) (12, 54) are reported less frequently (Figure 2).

### 3.4 Functions and effects of AI applications

#### 3.4.1 Prediction of risks and complications

**Figure 3:**
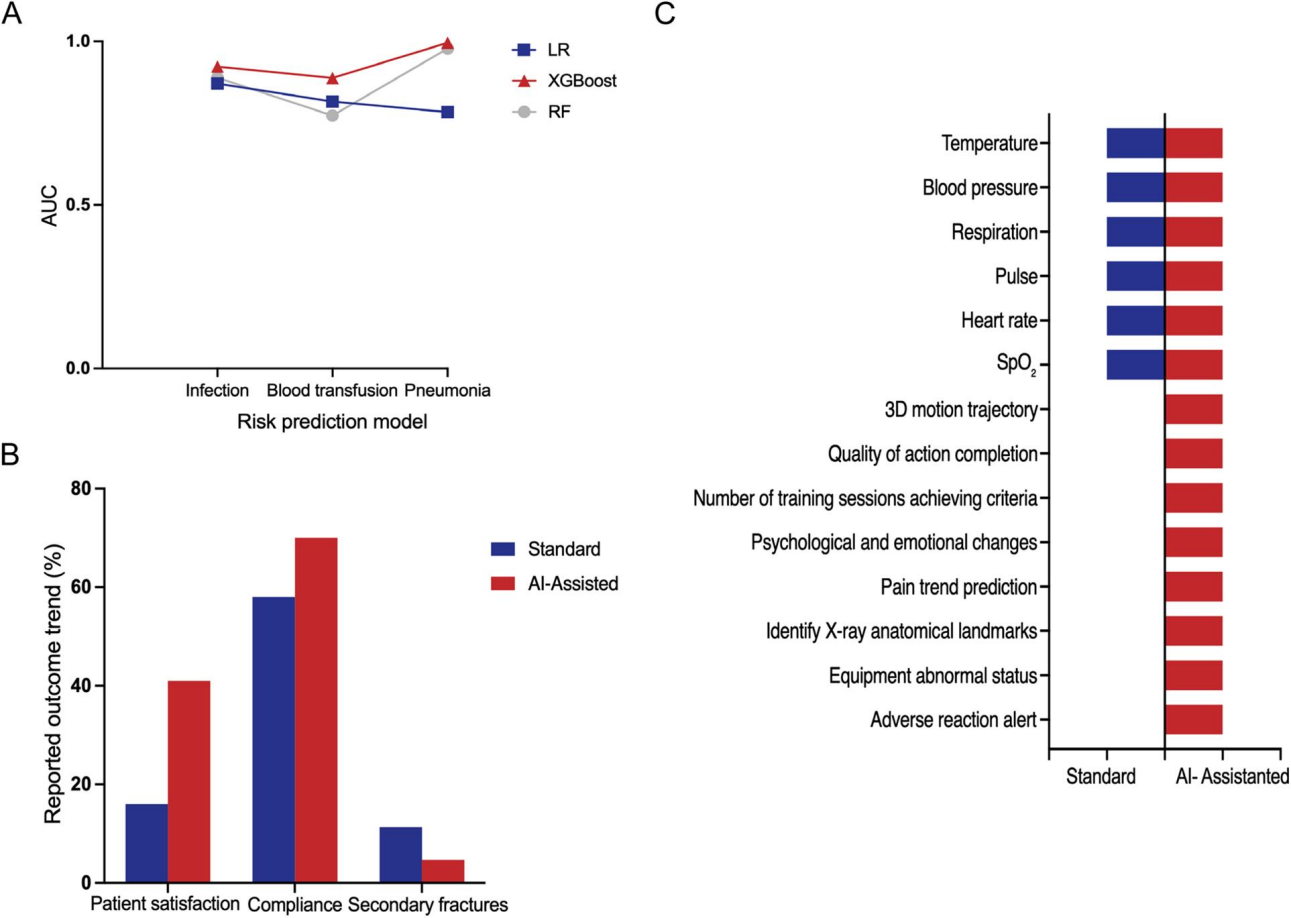
**(A)** Representative AUC values reported in different studies for postoperative risk prediction models based on logistic regression (logR), XGboost, and random forest (RF), including infection, blood transfusion, and pneumonia outcomes. Data were extracted from independent studies with different cohorts and are shown for illustrative comparison rather than direct algorithm benchmarking (10, 22, 26). **(B)** Representative outcome rates reported in selected studies comparing standard rehabilitation and AI-assisted rehabilitation. Values were extracted from individual studies with different sample sizes and outcome definitions and are presented to illustrate general trends rather than pooled estimates. **(C)** Comparison of measurable clinical and rehabilitation-related parameters that can be captured or monitored under standard rehabilitation vs. AI-assisted rehabilitation, based on synthesis of the included studies (presence indicates reported capability rather than quantitative superiority).

Numerous studies investigate the causal relationships between patients’ preoperative and/or intraoperative characteristics and postoperative outcomes, identifying factors associated with recovery, complication rates, and survival (10, 20–22, 24–28, 32, 33, 35–37, 41–46, 48, 51). For example, the primary determinants of gait speed changes following TKA are preoperative gait speed, age, and mechanical axis angle (43). Preoperative high-dose opioid use, postoperative dose escalation, and prolonged duration of use are identified as significant risk factors for long-term opioid dependence (37). After orthopedic surgery, there is often a risk of infection, pneumonia, and the need for blood transfusion. Therefore, some studies have applied ML algorithms to develop corresponding prediction models and used the area under the curve (AUC) value to assess model performance. The AUC value ranges from 0 to 1, where an AUC of 0.5 indicates no discriminatory power, equivalent to random guessing. For a model to have predictive value, its AUC should exceed 0.5, with values closer to 1 reflecting superior discriminative performance (55). Related research has evaluated the predictive capabilities of models built using algorithms such as LogR, eXtreme Gradient Boosting (XGBoost), and RF for these outcomes (10, 22, 26). The XGBoost model demonstrates the highest predictive accuracy for pneumonia risk, achieving an AUC value of 0.99. Similarly, the LogR algorithm shows strong performance in predicting infection, with an AUC value of 0.87. In contrast, the RF model exhibits a relatively lower AUC value of 0.77 when predicting blood transfusion needs (Figure 3A). This indicates that no single algorithm is optimal for all prediction tasks. Multiple algorithms must be compared and validated for different outcomes. It should be noted that these AUC values were extracted from different independent studies with distinct datasets and outcome definitions, and are presented here for illustrative comparison rather than direct head-to-head benchmarking of algorithms.

#### 3.4.2 Rehabilitation training and movement recognition

AI can assist in the standardized assessment of rehabilitation exercises by providing real-time feedback on both range of motion and movement quality. The TR systems enable real-time acquisition of patients’ limb movements data during exercises and correct non-standard movements through a combination of pose recognition and motion trajectory analysis (11, 12, 47). Furthermore, by leveraging NLP technologies such as chatbots, AI can deliver real-time responses to patient inquiries and offer personalized reminders, encouragement, and guidance in multiple formats (e.g., text, voice, and video), thereby enhancing patients’ health beliefs and adherence (30, 32). In addition, AI systems can automatically alert healthcare teams when abnormalities are detected, enabling clinical interventions (29, 31). Among the rehabilitation systems examined, the AI-assisted rehabilitation mode exhibits significantly higher patient satisfaction and compliance, as well as a lower incidence of secondary fractures compared with standard rehabilitation methods (Figure 3B).

#### 3.4.3 Assessment of rehabilitation progress and dynamic monitoring

Smart systems have significantly improved postoperative rehabilitation management by offering a broader and more detailed set of measurable parameters. It far surpassing the capabilities of traditional rehabilitation methods. Conventional rehabilitation models primarily focus on basic vital signs, such as body temperature, blood pressure, respiration rate, pulse, heart rate, and oxygen saturation (SpO_2_). In contrast, AI-assisted rehabilitation systems monitor a more comprehensive spectrum of indicators, encompassing not only traditional vital signs but also advanced parameters such as three-dimensional (3D) motion trajectories and the quality of action completion. In addition, they capture detailed information on the number of training sessions meeting performance criteria, psychological and emotional fluctuations, pain trend prediction, x-ray anatomical landmarks identification, equipment abnormal status alerts, and adverse reaction monitoring (Figure 3C). The shift from standard to AI-Assisted rehabilitation models has enhanced the effectiveness of treatment plans and improved the quality of life of patients. NLP systems actively engage patients by inquiring about pain intensity, wound conditions, and exercise adherence, allowing healthcare teams to monitor recovery progression and thereby improve treatment compliance and overall quality of life (52, 54). The implementation of intelligent pain management systems enables real-time monitoring of medical devices and medication usage, optimizes postoperative analgesia strategies, and effectively alleviates surgical pain (29, 31). For motor function assessment, remote intelligent systems dynamically evaluate training quality by continuously tracking patients’ movement data, thus contributing to improvements in joint function scores (11, 12). Additionally, AI algorithms based on image analysis continuously processed postoperative imaging data to monitor prosthesis positioning and joint alignment, achieving an accuracy rate exceeding 95% (50).

#### 3.4.4 Recommendation of individualized rehabilitation regimens

Through the analysis of heterogeneous data sources, AI enabled the formulation of individualized rehabilitation regimens and their dynamic adjustment, therefore improving the overall quality of care. By employing image segmentation with feature extraction, AI provided clinical decision support for identifying postoperative complications and generating nursing recommendations, assisting clinicians in developing more precise and timely interventions. Study results showed that patients in the intervention group exhibited superior joint function and activities of daily living, accompanied by significantly lower visual analogue scale (VAS) scores and reduced complication rates (23). Based on expert knowledge bases, healthcare databases, and individual patient data, AI generated personalized medication instructions and exercise protocols, which were continuously optimized according to the recovery trajectory. This approach facilitated patient self-management throughout rehabilitation, resulting in reduced postoperative pain and improved symptoms of anxiety and depression (12, 47). Furthermore, interactive chatbot-based systems collected patient inquiries, recovery progress, and psychological data, which were uploaded to healthcare databases for analysis and iterative updating by medical teams to optimize subsequent care delivery (30). Additionally, by integrating multimodal data such as demographic characteristics and laboratory results, AI constructed dynamic patient profiles that enabled comprehensive visualization of health status changes. These systems offered healthcare professionals with a holistic understanding of patient conditions, intelligently recommended tailored interventions and educational content, and ultimately improved self-efficacy, functional independence, and overall quality of life (34).

## 4 Discussion

### 4.1 Emphasize the external validation and clinical calibration of models

Notably, among the studies included in this scoping review, most models were developed and evaluated using single-center retrospective datasets, and only a very limited number reported external validation or calibration metrics beyond discrimination performance. The findings of this study show that ML is currently the most widely applied and one of the more mature branches of AI technologies in postoperative orthopedic rehabilitation. ML encompasses supervised, unsupervised, semi-supervised, and reinforcement learning. Through leveraged algorithms and statistical models, ML identifies patterns in clinical data, enabling predictions and informed decision-making for new or unknown data (56). Studies show that ML-based predictive models perform well in identifying high-risk patients for conditions such as surgical site infection (SSI) (10), deep vein thrombosis (DVT) (24), and delirium (25). Their AUC values could reach above 0.8. However, model performance depends on the quality of the data used and the methods of analysis and processing (57). For example, in studies predicting postoperative pneumonia, different models within the same research could show significant variation in AUC values, ranging from 0.81 to over 0.99. This suggests that during model development and application, it is essential to guard against the risk of overfitting and to emphasize external validation and clinical calibration (e.g., Brier score) to ensure robustness and practical utility, rather than blindly pursuing high AUC values.

### 4.2 Advance from predictive analysis to preventive intervention

AI technology holds the potential to drive effective clinical interventions. For example, AI can provide postoperative follow-up and personalized guidance to patients through standardized text messages and chatbots. Dwyer et al. (54) found that 79% of patients had their postoperative issues resolved through chatbots, and 48% did not seek additional medical help due to reassurance. Among 10 cases involving complication risks, 7 cases were properly managed, thus preventing overtreatment and unnecessary resource consumption. But this scoping review found that current research primarily focuses on model development and predictive performance validation. Specifically, the majority of included studies stopped at risk stratification or alert generation, while only a small subset translated prediction results into structured rehabilitation interventions or evaluated their impact on clinical outcomes. This limitation means that most predictive models serving merely as risk alerts, failing to integrate effectively with subsequent clinical actions. Therefore, future research should advance “predictive analysis” toward “preventive intervention”. For instance, based on the risk stratification generated by predictive models, tiered intervention strategies can be developed to implement enhanced rehabilitation training in advance. At the same time, compare the effectiveness differences between AI-assisted rehabilitation programs and traditional rehabilitation programs. This will validate the role of AI in rehabilitation outcomes.

### 4.3 Limited clinical translation and the need for higher-level evidence

Currently, the clinical translation of AI in postoperative orthopedic rehabilitation remains limited. Only a few examples, like ACS-National Surgical Quality Improvement Program (ACS-NSQIP) and X-TKA imaging analysis tools, have achieved preliminary translation. Within the reviewed literature, only a handful of studies reported real-world deployment or prospective evaluation, whereas most remained at the proof-of-concept or retrospective validation stage. Additionally, the evidence level of existing research is generally low, predominantly based on small-sample retrospective studies. Furthermore, the covered patient populations are relatively narrow, mainly focusing on those undergoing joint replacement and fracture surgeries. This limitation is partly due to the lengthy clinical validation cycles and the difficulty of accumulating sufficient evidence in a short period. For instance, the efficacy of arthroscopic surgery outcomes and SCI rehabilitation often requires months to years of follow-up, which constrains the thorough validation of AI prediction and decision support effectiveness. Future research should enhance clinical translation, conduct large-scale, multi-center, long-term follow-up clinical studies to enhance the evidence level and expand the scope of application to broader patient populations.

### 4.4 Strengthen privacy security and protect patient rights

Medical data often contains highly sensitive patient information. Data breaches can severely compromise patient privacy and expose individuals to risks such as discrimination or fraud, particularly in remote rehabilitation and cross-platform data sharing scenarios (58). In this scoping review, few studies explicitly reported data governance strategies, consent mechanisms, or privacy-preserving techniques, despite the frequent involvement of wearable devices and remote rehabilitation platforms. Therefore, AI applications must implement strict data security, storage and processing protocols. Simultaneously, fairness and ethical considerations should guide clinical trial design, resource allocation, and data collection to protect patient rights and well-being.

### 4.5 Transformation and collaboration of roles between healthcare professionals and patients

Across the included studies, AI systems were consistently positioned as decision support tools rather than autonomous decision makers, underscoring the necessity of human-AI collaboration in postoperative rehabilitation. The application of AI technology in postoperative orthopedic rehabilitation is driving a transformation of traditional rehabilitation models. The professional role of healthcare staff is gradually shifting from mere implementers to “human-machine collaborative decision-makers”. Meanwhile, patients were evolving from passive recipients of care to active participants in the rehabilitation process. A new collaborative relationship between healthcare professionals and patients, facilitated by AI, can be characterized in the following aspects. ① Implementation and optimization of rehabilitation plans. Although AI can generate individualized rehabilitation plans, clinicians are responsible for dynamically adjusting and refining interventions based on their professional judgment and the patient's actual condition. This approach ensures that care is safe and appropriate, rather than mechanically following algorithmic recommendations. ② Safety oversight in human-computer interaction. AI systems may exhibit data biases or algorithmic limitations. Jabbour et al. (59) reported that when an AI model contains systematic bias, clinician's diagnostic accuracy decreased by 11%. Consequently, healthcare professionals play a critical oversight role, continuously monitoring patient recovery, identifying AI system deficiencies, and mitigating their impact, thereby safeguarding both the safety and efficacy of rehabilitation. ③ Custodians of the humanistic connection. While AI can provide standardized guidance and informational feedback, it cannot replace the emotional support and humanistic care provided by healthcare professionals. Studies indicate that patients’ need for emotional support and interpersonal interaction remains unchanged despite AI integration (60). Through tactile comfort, verbal encouragement, and empathetic engagement, healthcare professionals help maintain patient trust and adherence throughout rehabilitation. ④ Transformation and collaboration of the patient's role. In AI-assisted rehabilitation, patients are active participants in their own recovery. Wearable devices and remote rehabilitation platforms enable patients to provide real-time feedback and engage in decision-making. Supported by both technological assistance and humanistic care, healthcare professionals collaborate with patients to establish and refine rehabilitation goals, forming a novel tripartite model of rehabilitation encompassing *technology, healthcare professionals, and patients*.

## 5 Conclusion

This scoping review systematically summarizes recent advancements in the application of AI in postoperative orthopedic rehabilitation. The findings suggest that AI shows promising potential in areas such as prediction, feedback, monitoring, decision support, and data acquisition, indicating emerging opportunities to improve rehabilitation outcomes and reducing the risk of complications. However, this scoping review also has some limitations. Restricting the search to Chinese and English publications, together with the over representation of studies from China, may limit the global generalizability of the results, as healthcare systems and rehabilitation practices vary across regions. At the same time, this distribution reflects the rapid development of AI-assisted rehabilitation in China, which may serve as an important testing ground for scalable models. Second, the analysis is largely descriptive and did not include a formal critical appraisal of the methodological quality or risk of bias in the included literatures. Future research should focus on elucidating the specific mechanisms underlying AI applications in postoperative orthopedic rehabilitation and conduct high-quality, multi-center clinical trials with large sample sizes. Such efforts will promote the deeper integration of AI technology with clinical rehabilitation, ultimately providing more scientific and personalized support for postoperative orthopedic recovery.
