# Supplementary material for: Application of artificial intelligence in postoperative orthopedic rehabilitation: a scoping review
Source: Front Digit Health. 2026 Jan 14;7:1746552. doi: 10.3389/fdgth.2025.1746552 (PMC12847308; doi:10.3389/fdgth.2025.1746552)
Supplement: Supplementary file 2 [file Datasheet2.pdf]

This is the search strategy in databases.

The search strategy for PubMed (2020-3 to 2025-3)

Search	Query
#1	"artificial intelligence"[Mesh] OR "data mining"[Mesh] OR "fuzzy logic" [Mesh] OR "neural networks, computer"[Mesh] OR "machine learning"[Mesh] OR "natural language processing"[Mesh]
#2	"computer reasoning"[Title/Abstract] OR "AI (Artificial Intelligence)"[Title/Abstract] OR "machine intelligence"[Title/Abstract] OR "computational intelligence"[Title/Abstract] OR "computer vision systems"[Title/Abstract] OR "computer vision system"[Title/Abstract] OR "knowledge representation"[Title/Abstract] OR "knowledge acquisition"[Title/Abstract] OR "fuzzy algorithms"[Title/Abstract] OR "neural network"[Title/Abstract] OR "computer neural network"[Title/Abstract] OR "neural network model"[Title/Abstract] OR "computational neural network"[Title/Abstract] OR "bayesian networks"[Title/Abstract] OR "text mining"[Title/Abstract] OR "deep learning"[Title/Abstract] OR "random forest"[Title/Abstract] OR "support vector"[Title/Abstract] OR "algorithms"[Title/Abstract] OR "expert systems"[Title/Abstract]
#3	orthopedics[Mesh]
#4	"fracture"[Title/Abstract] OR "arthroplasty, replacement"[Title/Abstract] OR "arthroplasty"[Title/Abstract] OR "joint"[Title/Abstract] OR "hip"[Title/Abstract] OR "knee"[Title/Abstract] OR "shoulder"[Title/Abstract] OR "ankle"[Title/Abstract] OR "wrist"[Title/Abstract] OR "elbow"[Title/Abstract] OR "finger"[Title/Abstract] OR "spine"[Title/Abstract] OR "spinal"[Title/Abstract] OR "vertebra"[Title/Abstract] OR "skeleton"[Title/Abstract] OR "dislocation"[Title/Abstract] OR "subluxation"[Title/Abstract] OR "trauma"[Title/Abstract] OR "arthritis"[Title/Abstract] OR "osteomyelitis"[Title/Abstract] OR "osteoporosis"[Title/Abstract] OR "ACL"[Title/Abstract] OR "PCL"[Title/Abstract] OR "bone tumor"[Title/Abstract] OR "bone neoplasms"[Title/Abstract]
#5	"postoperative period"[Mesh] OR "postoperative care"[Mesh] OR "rehabilitation"[Mesh] OR "recovery of function"[Mesh]
#6	"postoperative periods"[Title/Abstract] OR "postoperative procedures"[Title/Abstract] OR "postoperative procedure"[Title/Abstract] OR "function recoveries"[Title/Abstract] OR "function recovery"[Title/Abstract] OR "postoperative rehabilitation"[Title/Abstract] OR "post-surgical recovery"[Title/Abstract] OR "surgical recovery"[Title/Abstract]
#7	(#1 OR #2) AND (#3 OR #4) AND (#5 OR #6)

The search strategy for CINAHL Complete (2020-3 to 2025-3)

Search	Query
S1	MH ((MH "Artificial Intelligence+") OR (MH "Data Mining+") OR (MH "Algorithms+") OR (MH "Neural Networks (Computer)+") OR (MH "Machine Learning+") OR (MH "Natural Language Processing")) OR TI ("computer reasoning" OR "machine intelligence" OR "computational intelligence" OR "computer vision systems" OR

	"computer vision system" OR "knowledge representation" OR "knowledge acquisition" OR "fuzzy logic" OR "fuzzy algorithms" OR "neural network" OR "computer neural network" OR "neural network model" OR "computational neural network" OR "bayesian networks" OR "text mining" OR "deep learning" OR "random forest" OR "support vector" OR "expert systems") OR AB ("computer reasoning" OR "machine intelligence" OR "computational intelligence" OR "computer vision systems" OR "computer vision system" OR "knowledge representation" OR "knowledge acquisition" OR "fuzzy logic" OR "fuzzy algorithms" OR "neural network" OR "computer neural network" OR "neural network model" OR "computational neural network" OR "bayesian networks" OR "text mining" OR "deep learning" OR "random forest" OR "support vector" OR "expert systems")
S2	MH (MH "Orthopedics") OR TI ("fracture" OR "arthroplasty, replacement" OR "arthroplasty" OR "joint" OR "hip" OR "knee" OR "shoulder" OR "ankle" OR "wrist" OR "elbow" OR "finger" OR "spine" OR "spinal" OR "vertebra" OR "skeleton" OR "dislocation" OR "subluxation" OR "trauma" OR "arthritis" OR "osteomyelitis" OR "osteoporosis" OR "ACL" OR "PCL" OR "bone tumor" OR "bone neoplasms") OR AB ("fracture" OR "arthroplasty, replacement" OR "arthroplasty" OR "joint" OR "hip" OR "knee" OR "shoulder" OR "ankle" OR "wrist" OR "elbow" OR "finger" OR "spine" OR "spinal" OR "vertebra" OR "skeleton" OR "dislocation" OR "subluxation" OR "trauma" OR "arthritis" OR "osteomyelitis" OR "osteoporosis" OR "ACL" OR "PCL" OR "bone tumor" OR "bone neoplasms")
S3	MH ((MH "Postoperative Period") OR (MH "Postoperative Care+") OR (MH "Rehabilitation+")) OR TI ("recovery of function" OR "postoperative periods" OR "postoperative procedures" OR "postoperative procedure" OR "function recoveries" OR "function recovery" OR "postoperative rehabilitation" OR "post-surgical recovery" OR "surgical recovery") OR AB ("recovery of function" OR "postoperative periods" OR "postoperative procedures" OR "postoperative procedure" OR "function recoveries" OR "function recovery" OR "postoperative rehabilitation" OR "post-surgical recovery" OR "surgical recovery")
S4	S1 AND S2 AND S3

The search strategy for The Cochrane Library (2020-3 to 2025-3)

Search	Query
#1	MeSH descriptor: [Artificial Intelligence] explode all trees
#2	MeSH descriptor: [Data Mining] explode all trees
#3	MeSH descriptor: [Fuzzy Logic] explode all trees
#4	MeSH descriptor: [Neural Networks, Computer] explode all trees
#5	MeSH descriptor: [Machine Learning] explode all trees
#6	MeSH descriptor: [Natural Language Processing] explode all trees
#7	('neural networks, computer' OR 'computer reasoning' OR 'machine intelligence' OR 'computational intelligence' OR 'computer vision systems' OR 'computer vision system' OR 'knowledge representation' OR 'knowledge acquisition' OR 'fuzzy algorithms' OR 'neural network' OR 'computer neural network' OR 'neural network model' OR 'computational neural network' OR 'bayesian networks' OR 'text mining' OR 'deep

	learning' OR 'random forest' OR 'support vector' OR 'algorithms' OR 'expert systems'):ti,ab,kw
#8	MeSH descriptor: [Orthopedics] explode all trees
#9	('fracture' OR 'arthroplasty, replacement' OR 'arthroplasty' OR 'joint' OR 'hip' OR 'knee' OR 'shoulder' OR 'ankle' OR 'wrist' OR 'elbow' OR 'finger' OR 'spine' OR 'spinal' OR 'vertebra' OR 'skeleton' OR 'dislocation' OR 'subluxation' OR 'trauma' OR 'arthritis' OR 'osteomyelitis' OR 'osteoporosis' OR 'ACL' OR 'PCL' OR 'bone tumor' OR 'bone neoplasms'):ti,ab,kw
#10	MeSH descriptor: [Postoperative Period] explode all trees
#11	MeSH descriptor: [Postoperative Care] explode all trees
#12	MeSH descriptor: [Rehabilitation] explode all trees
#13	MeSH descriptor: [Recovery of Function] explode all trees
#14	('postoperative periods' OR 'postoperative procedures' OR 'postoperative procedure' OR 'function recoveries' OR 'function recovery' OR 'postoperative rehabilitation' OR 'post-surgical recovery' OR 'surgical recovery'):ti,ab,kw
#15	#1 OR #2 OR #3 OR #4 OR #5 OR #6 OR #7
#16	#8 OR #9
#17	#10 OR #11 OR #12 OR #13 OR #14
#18	#15 AND #16 AND #17

The search strategy for Web of Science (2020-3 to 2025-3)

Search	Query
#1	TS=("artificial intelligence" OR "data mining" OR "fuzzy logic" OR "neural networks, computer" OR "machine learning" OR "natural language processing" OR "computer reasoning" OR "machine intelligence" OR "computational intelligence" OR "computer vision systems" OR "computer vision system" OR "knowledge representation" OR "knowledge acquisition" OR "fuzzy algorithms" OR "neural network" OR "computer neural network" OR "neural network model" OR "computational neural network" OR "bayesian networks" OR "text mining" OR "deep learning" OR "random forest" OR "support vector" OR "algorithms" OR "expert systems")
#2	TS=("orthopedics" OR "fracture" OR "arthroplasty, replacement" OR "arthroplasty" OR "joint" OR "hip" OR "knee" OR "shoulder" OR "ankle" OR "wrist" OR "elbow" OR "finger" OR "spine" OR "spinal" OR "vertebra" OR "skeleton" OR "dislocation" OR "subluxation" OR "trauma" OR "arthritis" OR "osteomyelitis" OR "osteoporosis" OR "ACL" OR "PCL" OR "bone tumor" OR "bone neoplasms")
#3	TS=("postoperative period" OR "postoperative care" OR "rehabilitation" OR "recovery of function" OR "postoperative periods" OR "postoperative procedures" OR "postoperative procedure" OR "function recoveries" OR "function recovery" OR "postoperative rehabilitation" OR "post-surgical recovery" OR "surgical recovery")
#4	#1 AND #2 AND #3

The search strategy for Embase (2020-3 to 2025-3)

Search	Query
#1	artificial intelligence'/exp OR 'data mining'/exp OR 'fuzzy logic'/exp OR 'artificial neural network'/exp OR 'machine learning'/exp OR 'natural language processing'/exp
#2	('neural networks, computer' OR 'computer reasoning' OR 'machine intelligence' OR 'computational intelligence' OR 'computer vision systems' OR 'computer vision system' OR 'knowledge representation' OR 'knowledge acquisition' OR 'fuzzy algorithms' OR 'neural network' OR 'computer neural network' OR 'neural network model' OR 'computational neural network' OR 'bayesian networks' OR 'text mining' OR 'deep learning' OR 'random forest' OR 'support vector' OR 'algorithms' OR 'expert systems'):ti,ab,kw
#3	orthopedics'/exp
#4	('fracture' OR 'arthroplasty, replacement' OR 'arthroplasty' OR 'joint' OR 'hip' OR 'knee' OR 'shoulder' OR 'ankle' OR 'wrist' OR 'elbow' OR 'finger' OR 'spine' OR 'spinal' OR 'vertebra' OR 'skeleton' OR 'dislocation' OR 'subluxation' OR 'trauma' OR 'arthritis' OR 'osteomyelitis' OR 'osteoporosis' OR 'ACL' OR 'PCL' OR 'bone tumor' OR 'bone neoplasms'):ti,ab,kw
#5	postoperative period'/exp OR 'rehabilitation'/exp OR 'postoperative care'/exp OR 'convalescence'/exp
#6	('recovery of function' OR 'postoperative periods' OR 'postoperative procedures' OR 'postoperative procedure' OR 'function recoveries' OR 'function recovery' OR 'postoperative rehabilitation' OR 'post-surgical recovery' OR 'surgical recovery'):ti,ab,kw
#7	#1 OR #2
#8	#3 OR #4
#9	#5 OR #6
#10	#7 AND #8 AND #9

The search strategy for Scopus (2020-3 to 2025-3)

Search	Query
#1	(TITLE-ABS-KEY ("postoperative period") OR TITLE-ABS-KEY ("postoperative care") OR TITLE-ABS-KEY ("rehabilitation") OR TITLE-ABS-KEY ("recovery of function") OR TITLE-ABS-KEY ("postoperative periods") OR TITLE-ABS-KEY ("postoperative procedures") OR TITLE-ABS-KEY ("postoperative procedure") OR TITLE-ABS-KEY ("function recoveries") OR TITLE-ABS-KEY ("function recovery") OR TITLE-ABS-KEY ("postoperative rehabilitation") OR TITLE-ABS-KEY ("post-surgical recovery") OR TITLE-ABS-KEY ("surgical recovery"))
#2	(TITLE-ABS-KEY ("orthopedics") OR TITLE-ABS-KEY ("fracture") OR TITLE-ABS-KEY ("arthroplasty, replacement") OR TITLE-ABS-KEY ("arthroplasty") OR TITLE-ABS-KEY ("joint") OR TITLE-ABS-KEY ("hip") OR TITLE-ABS-KEY ("knee") OR TITLE-ABS-KEY ("shoulder") OR TITLE-ABS-KEY ("ankle") OR TITLE-ABS-KEY ("wrist") OR TITLE-ABS-KEY ("elbow") OR TITLE-ABS-KEY ("finger") OR TITLE-ABS-KEY ("spine") OR TITLE-ABS-KEY ("spinal") OR TITLE-ABS-KEY ("vertebra") OR TITLE-ABS-KEY ("skeleton") OR TITLE-ABS-KEY ("dislocation") OR TITLE-ABS-KEY ("subluxation") OR TITLE-ABS-KEY

	("trauma") OR TITLE-ABS-KEY ("arthritis") OR TITLE-ABS-KEY ("osteomyelitis") OR TITLE-ABS-KEY ("osteoporosis") OR TITLE-ABS-KEY ("ACL") OR TITLE-ABS-KEY ("PCL") TITLE-ABS-KEY ("bone tumor") OR TITLE-ABS-KEY ("bone neoplasms"))
#3	(TITLE-ABS-KEY ("artificial intelligence") OR TITLE-ABS-KEY ("data mining") OR TITLE-ABS-KEY ("fuzzy logic") OR TITLE-ABS-KEY ("neural networks, computer") OR TITLE-ABS-KEY ("machine learning") OR TITLE-ABS-KEY ("natural language processing") OR TITLE-ABS-KEY ("computer reasoning") OR TITLE-ABS-KEY ("machine intelligence") OR TITLE-ABS-KEY ("computational intelligence") OR TITLE-ABS-KEY ("computer vision systems") OR TITLE-ABS-KEY ("computer vision system") OR TITLE-ABS-KEY ("knowledge representation") OR TITLE-ABS-KEY ("knowledge acquisition") OR TITLE-ABS-KEY ("fuzzy algorithms") OR TITLE-ABS-KEY ("neural network") OR TITLE-ABS-KEY ("computer neural network") OR TITLE-ABS-KEY ("neural network model") OR TITLE-ABS-KEY ("computational neural network") OR TITLE-ABS-KEY ("bayesian networks") OR TITLE-ABS-KEY ("text mining") OR TITLE-ABS-KEY ("deep learning") OR TITLE-ABS-KEY ("random forest") OR TITLE-ABS-KEY ("support vector") OR TITLE-ABS-KEY ("algorithms") OR TITLE-ABS-KEY ("expert systems"))
#4	#1 AND #2 AND #3

The search strategy for IEEE Xplore (2020-3 to 2025-3)

Search	Query
#1	("All Metadata":"artificial intelligence" OR "All Metadata":"data mining" OR "All Metadata":"fuzzy logic" OR "All Metadata":"neural networks, computer" OR "All Metadata":"machine learning" OR "All Metadata":"natural language processing" OR "All Metadata":"computer reasoning" OR "All Metadata":"machine intelligence" OR "All Metadata":"computational intelligence" OR "All Metadata":"computer vision systems" OR "All Metadata":"computer vision system" OR "All Metadata":"knowledge representation" OR "All Metadata":"knowledge acquisition" OR "All Metadata":"fuzzy algorithms" OR "All Metadata":"neural network" OR "All Metadata":"computer neural network" OR "All Metadata":"neural network model" OR "All Metadata":"computational neural network" OR "All Metadata":"bayesian networks" OR "All Metadata":"text mining" OR "All Metadata":"deep learning" OR "All Metadata":"random forest" OR "All Metadata":"support vector" OR "All Metadata":"algorithms" OR "All Metadata":"expert systems")
#2	("All Metadata":"orthopedics" OR "All Metadata":"fracture" OR "All Metadata":"arthroplasty, replacement" OR "All Metadata":"arthroplasty" OR "All Metadata":"joint" OR "All Metadata":"hip" OR "All Metadata":"knee" OR "All Metadata":"shoulder" OR "All Metadata":"ankle" OR "All Metadata":"wrist" OR "All Metadata":"elbow" OR "All Metadata":"finger" OR "All Metadata":"spine" OR "All Metadata":"spinal" OR "All Metadata":"vertebra" OR "All Metadata":"skeleton" OR "All Metadata":"dislocation" OR "All Metadata":"subluxation" OR "All Metadata":"trauma" OR "All Metadata":"arthritis" OR "All Metadata":"osteomyelitis" OR "All

	Metadata":"osteoporosis" OR "All Metadata":"ACL" OR "All Metadata":"PCL" OR "All Metadata":"bone tumor" OR "All Metadata":"bone neoplasms")
#3	("All Metadata":"postoperative period" OR "All Metadata":"postoperative care" OR "All Metadata":"rehabilitation" OR "All Metadata":"recovery of function" OR "All Metadata":"postoperative periods" OR "All Metadata":"postoperative procedures" OR "All Metadata":"postoperative procedure" OR "All Metadata":"function recoveries" OR "All Metadata":"function recovery" OR "All Metadata":"postoperative rehabilitation" OR "All Metadata":"post-surgical recovery" OR "All Metadata":"surgical recovery")
#4	#1 AND #2 AND #3

The search strategy for SinoMed (2020-3 to 2025-3)

Search	Query
#1	"人工智能"[不加权:扩展]
#2	"数据挖掘"[常用字段:智能] OR "贝叶斯网络"[常用字段:智能] OR "文本挖掘"[常用字段:智能] OR "深度学习"[常用字段:智能] OR "非自然语言处理"[常用字段:智能] OR "随机森林"[常用字段:智能] OR "支持向量机"[常用字段:智能]
#3	"骨科"[常用字段:智能] OR "骨折"[常用字段:智能] OR "骨头"[常用字段:智能] OR "*关节"[常用字段:智能] OR "关节置换"[常用字段:智能] OR "脊*"[常用字段:智能] OR "*椎"[常用字段:智能] OR "脱位"[常用字段:智能] OR "韧带重建"[常用字段:智能]
#4	"关节炎"[常用字段:智能] OR "骨髓炎"[常用字段:智能] OR "骨质疏松"[常用字段:智能] OR "骨*瘤"[常用字段:智能]
#5	"术后"[常用字段:智能] OR "康复"[常用字段:智能] OR "恢复"[常用字段:智能] OR "术后康复"[常用字段:智能] OR "术后护理"[常用字段:智能] OR "功能恢复"[常用字段:智能] OR "机能恢复"[常用字段:智能] OR "康复训练"[常用字段:智能]
#6	(#2) OR (#1)
#7	(#4) OR (#3)
#8	(#7) AND (#6) AND (#5)

不加权:扩展, unweighted: expansion

常用字段:智能, common fields: intelligent

The search strategy for CNKI (2020-3 to 2025-3)

Search	Query
#1	SU=(人工智能 + 机器智能 + 数据挖掘 + 模糊算法 + 神经网络 + 贝叶斯网络 + 文本挖掘 + 模糊逻辑 + 知识表达 + 机器学习 + 深度学习 + 自然语言处理 + 非自然语言处理 + 随机森林 + 支持向量机 + 算法)
#2	SU=(骨科 + 骨折 + 骨头 +*关节 + 关节置换 + 脊柱 + 脊髓 +*椎 + 脱位 +*伤 + 关节炎 + 骨髓炎 + 骨质疏松 + 韧带重建 + 骨*瘤)
#3	SU=(术后 + 康复 + 恢复 + 术后康复 + 术后护理 + 功能恢复 + 机能恢复 + 康复训练)
#4	#1 AND #2 AND #3

SU, Topic

The search strategy for WanFang Database (2020-3 to 2025-3)

Search	Query
#1	(主题:("人工智能" OR "机器智能" OR "数据挖掘" OR "模糊算法" OR "神经网络" OR "贝叶斯网络" OR "文本挖掘" OR "模糊逻辑" OR "知识表达" OR "机器学习" OR "深度学习" OR "自然语言处理" OR "非自然语言处理" OR "随机森林" OR "支持向量机" OR "算法"))
#2	(主题:("骨科" OR "骨折" OR "骨头" OR "*关节" OR "关节置换" OR "脊柱" OR "脊髓" OR "*椎" OR "脱位" OR "关节炎" OR "骨髓炎" OR "骨质疏松" OR "韧带重建" OR "骨*瘤"))
#3	(主题:(术后 OR 康复 OR 恢复 OR 术后康复 OR 术后护理 OR 功能恢复 OR 机能恢复 OR 康复训练))
#4	#1 AND #2 AND #3

主题, Topic
